# Editorial: Digital Solutions in Cardiology

**DOI:** 10.3389/fcvm.2022.873991

**Published:** 2022-04-08

**Authors:** Mark J. Schuuring, Alexandru N. Mischie, Enrico G. Caiani

**Affiliations:** ^1^Department of Cardiology, Amsterdam University Medical Centers, University of Amsterdam, Amsterdam, Netherlands; ^2^Centre Hospitalier Montlucon, Department of Cardiology, Montluçon, France; ^3^International Society of Telemedicine and eHealth, Montluçon, France; ^4^Politecnico di Milano, Department of Electronics, Information and Biomedical Engineering, Milan, Italy; ^5^National Council of Research, Institute of Electronics, Information and Telecommunication Engineering, Milan, Italy

**Keywords:** artificial intelligence, telemedicine, cardiology, cardiovascular abnormalities, ACHD, transcatheter aortic valve implantation, heart diseases, cardiac imaging

Cardiovascular disease is the most common cause of morbidity and mortality worldwide (1). To make an effort to contribute to the reduction of morbidity and mortality the guest associate editors have started a Research Topic so that knowledge about a number of unique digital solutions became available. Digital solutions are promising for monitoring patients, relieving patients and doctors from work and care and for early detection and intervention (2–5). This special issue covers specific areas and application of digital solutions in cardiology, including digital education, virtual care organization, a digital decision support system, a virtual modeling, and solutions in the field of machine learning.

The coronavirus disease-2019 (COVID-19) pandemic has led to significant disruption with subsequent innovation and acceleration of digital solutions (6, 7). Chong et al. performed a review on digitalization of cardiovascular training and education during this pandemic. The maturation of technological infrastructure for acute and chronic remote cardiac care provision improved e-learning capabilities for trainees. The authors describe technology-enabled learning solutions, associated infrastructure needs, adoption, and governance. The advantages of these digital solutions can be leveraged in cardiovascular medicine and wider medical education to provide effective, inclusive, and equitable training of doctors through the current pandemic and beyond.

A concept of a virtual care center (VCC) has been presented by Van der Lande. This VCC integrates first, second-, and third-line care into a virtual ward using remote monitoring and video consultation. The authors designed this VCC for patients diagnosed with three or more chronic conditions. These patients receive remote monitoring and video consultations on smartphone compatible devices. Follow-up will be performed by the VCC, consisting of nurses who coordinate care, supervised by general practitioners and medical specialists. Data will be reviewed on a daily basis and patients will be contacted on a weekly basis. Review of data is automated by computer algorithms. Patients will be contacted in case of outcome abnormalities in the data. Patients can contact the VCC at any time. Follow-up of this digital solution is 1 year, and the primary outcome of this study is the median number of nights admitted to the hospital per patient compared to the hospitalization data 12 months before enrolment. Secondary, outcomes include all-cause mortality, event free survival, quality of life, and satisfaction with technology and care. Virtual wards are discussed in this paper and results are awaited.

The next paper focusses on a clinical decision support system as a digital solution in congenital heart disease (CHD) patients (Assadi et al.). This group of young patients with CHD seem particularly suited for digital solutions (8). There are differences in the cognitive processes used by CHD experts and emergency department (ED) physicians when managing CHD patients. An understanding of differences in the cognitive processes used by CHD experts and ED physicians can inform the development of potential interventions, such as clinical decision support systems and training pathways, to support decision making pertaining to the acute treatment of CHD patients.

Brenneisen et al. described a digital solution on virtual modeling *in silico* to reproduce physiological characteristics and diseases of the heart. Particularly the simulation of the blood hemodynamics and its interaction with the myocardial tissue remains complex. The authors suggested a cycle-to-cycle coupling of the structural deformation and the fluid dynamics. This appeared to be a promising approach to account for this fluid-structure interaction with low computational effort. In an individualized healthy whole-heart model, one iteration sufficed to obtain converged and physiologically plausible results.

Machine learning (ML), an advanced digital solutions, has a tremendous potential impact on (interventional) cardiology (9, 10). Lopes et al. presented original research on outcome prediction in patients who undergo transcatheter aortic valve implantation (TAVI). This study shows that distributed ML and combined local model techniques, can overcome data sharing limitations and result in more accurate models for TAVI mortality estimation. The authors have shown improved prognostic accuracy for both centers and can also be used as an alternative to overcome the problem of limited amounts of data when creating prognostic models.

In conclusion, this Research Topic covers a number of unique digital solutions in the field of cardiology, and now it is up to the readers to start using these digital solutions. Implementation of digital solutions can be challenge, however, numerous papers are available to help overcome challenges (6, 11). The time for digital solutions is therefore now!

## Author Contributions

All authors listed have made a substantial, direct, and intellectual contribution to the work and approved it for publication.

## Conflict of Interest

The authors declare that the research was conducted in the absence of any commercial or financial relationships that could be construed as a potential conflict of interest.

## Publisher's Note

All claims expressed in this article are solely those of the authors and do not necessarily represent those of their affiliated organizations, or those of the publisher, the editors and the reviewers. Any product that may be evaluated in this article, or claim that may be made by its manufacturer, is not guaranteed or endorsed by the publisher.
